# The Impact of Recent Generic Drug Price Policies on Pharmaceutical Innovation: A Theoretical Rationale and Proposal of a Method Supporting Innovation in Areas of Unmet Medical Need

**DOI:** 10.36469/9838

**Published:** 2016-10-27

**Authors:** Pierre-Alexandre Dionne, Farzad Ali, Mendel Grobler

**Affiliations:** 1 Pfizer Canada Inc., Kirkland, Canada and Department of Community Health Services, University of Sherbrooke, Longueuil, Canada; 2 Pfizer Canada Inc., Kirkland, Canada; 3 Pfizer Australia Inc., West Ryde, Australia

**Keywords:** pharmaceutical innovation, unmet medical needs, drug price regulation, cost-effectiveness analysis, health technology assessment

## Abstract

New discoveries are a critical priority for the pharmaceutical industry. However, the use of fixed incremental cost-effectiveness (ICER) thresholds for health technology assessment (HTA) may compromise incentives to innovate and affect future treatment options. This paper highlights the impact of generic drug price policies on pharmaceutical innovation in the context of fixed ICER thresholds and proposes a new consideration for the cost-effectiveness analysis (CEA).

There is a direct causal relationship between HTA and the market price of a drug; in jurisdictions where HTA agencies apply fixed ICER thresholds as an important reimbursement listing criterion, the incremental cost of a new drug is expected to be proportional to its incremental benefit over the comparator. However, the comparator price is subject to market forces or sudden policies and may change markedly affecting the cost-effectiveness assessment (e.g. where the comparator patent has expired). Since recent generic price regulations increased the price gap between drugs’ generic and patented versions, it is harder to achieve a sufficient level of incremental benefits in order to offset incremental prices of new treatments. Consequently, even promising drugs may have challenges to show attractive ICERs and research and development (R&D) investments may become unattractive in certain disease area.

In order to promote innovation in therapeutic fields with unmet medical needs, a compromise would be to include the comparator’s patented price in the CEA instead of the generic drug. By identifying the relevant disease areas, decision makers and HTA authorities could therefore convey the importance of investing in these therapeutic areas to manufacturers.

## INTRODUCTION

Similar to many countries around the globe, the HTA process is widely used by national and provincial bodies in Canada. While assessment of new drugs for most of Canada is performed by the Canadian Agency for Drug and Technologies in Health (CADTH), generic price policies and drug coverage vary among the 10 Canadian provinces.[1,2] For example, in Ontario, the largest drug market in Canada, new generic price regulations have been adopted to force drug manufacturers to lower the generic price to 25% of the patented drug price.[3] In addition, Ontario's government recently announced its plans to lower the regulated generic price for the top 10 generic drugs on the public formulary, from 25% to 20% of the branded price.[4] Even though these price regulations are very relevant to restrain public drug spending, no measures are taken to adapt HTA frameworks to reflect these changes in Canada, where the incremental cost-effectiveness ratio (ICER) of a new drug appears to play an important role in decision-making.[5] Indirectly, these price regulations combined with the use of fixed ICER threshold in the HTA process may impact research and development (R&D) investments by pharmaceutical firms and future innovation for patients' health.

This article first discusses pharmaceutical innovation, the impact of recent changes in generic prices, and the utilization of fixed ICER thresholds by HTA agencies. Second, we propose a new methodological aspect that could be considered in the cost-effectiveness analysis (CEA) of certain drugs in order to promote innovation in areas of unmet needs.

### Pharmaceutical Innovation and Incentives

Innovation is a critical priority for the pharmaceutical industry, in which the primary aim is to address unmet medical needs. Therefore, research-oriented pharmaceutical companies invest a sizeable portion of their revenues on R&D, which translates into innovation that ultimately increases life expectancy and quality of life.[6,7] To promote this innovation, the patent system grants the manufacturer exclusivity in the commercialization of a new drug for an allotted period of time and permits attractive returns on investment (ROI) that will be in turn partly invested into R&D.[7,8] A high ROI stimulates innovation and will ultimately be beneficial for future patients.[9]

On the other hand, the health technology assessment (HTA) using cost-effectiveness (CE) threshold as an important criterion for public drug reimbursement may decrease these incentives. Although HTA is intended to result in a better and fairer allocation of health care resources in the context of limited budgets in several countries with national payers such as the United Kingdom, Australia, and Canada[10], it results in various drugs being without public reimbursement in some therapeutic areas such as oncology and Alzheimer.[11,12] Obtaining reimbursement coverage has become an important concern for research-based pharmaceutical firms to the extent that it may affect R&D decisions and have extensive consequences.[13] The question is then to which point the notion of static allocative efficiency (i.e. the strict use of CE criteria and price regulations that focus only on short-term cost reductions) is monetary and morally acceptable considering the related reduction in incentives for future innovation.[14]

### The Use of Cost-effectiveness Thresholds for Drug Reimbursement Purposes

The use of HTA has been growing rapidly around the globe. Considering the aging of the population and the rapid increase in the cost of drugs and medical technologies, its use has become highly relevant in ensuring a better allocation of resources.[10] Consequently, the economic value of a new drug or technology has become a widely accepted requirement for public reimbursement.[10,15] This requirement has been labelled as "the fourth hurdle": along with quality, efficacy, and safety, it is necessary to demonstrate cost-effectiveness to ensure market access of a new technology. The economic value is generally measured using an incremental cost-effectiveness ratio (ICER), which represents the ratio between the difference in costs and the difference in benefits of a new drug as compared with the standard of care or another comparator drug. For the purpose of cost-utility analyses (CUA), which is the most frequently used cost-effectiveness analysis for drug assessment, the ICER is interpreted using a cost per quality-adjusted life year (QALY), which takes into account the effect on both life expectancy and quality of life.[16]

Although the use of the QALY was initiated for the purpose of comparing different technologies on the same result unit and was supposed to be an arbitrary figure, some authors have reported the use of fixed ICER thresholds by HTA agencies to determine the acceptability of new technologies or new drugs.[17-19] According to previous studies, these thresholds would be approximately CAD$50,000 in Canada, AUD$42,000 in Australia and £30,000 in the United Kingdom.[17-19] ICER thresholds have incorrectly been related to historically accepted levels of efficiency, such as the ICER of end-stage renal disease kidney dialysis in the 1970s or 1980s, and remain an arbitrary concept without strong empirical foundation.[19]

Due to the innovation and the associated commercialization of new agents of the same class, the clinical effectiveness of standards of care is expected to increase over time within a disease area.[14] After successive incremental innovations in a given therapeutic class, the effectiveness of a new drug tends to equal the clinical effectiveness ceiling (Ed max) for this specific disease.[20] Ed max thus corresponds to a theoretical ideal in a given disease area in order to completely cure the disease or allow patients to live without major disabilities. In most disease areas, Ed max is only achievable with successive incremental innovations; however, under a fixed ICER threshold, the incremental effectiveness of a new drug is expected to be lower over time, which effectively translates into a lower incremental price as well.[14] Although it is logical and purposeful to require a new drug to show significant incremental benefits to justify the difference in price, HTA using fixed ICER thresholds may result in some issues in certain therapeutic classes.

## The Need for an Improved Decision-making Process for the Reimbursement of Innovative Pharmacotherapy

In therapeutic areas presenting unmet medical needs (i.e. where Ed max is far from being reached) the use of a fixed acceptability ICER threshold for the reimbursement listing of innovative drugs may not contribute to the enhancement of innovation in the drug development process. New generations of treatments have high acquisition prices, illustrating high R&D costs and relatively small target populations.[21,22] Although many of these treatments demonstrate promising clinical outcomes, their cost-effectiveness incremental ratios (ICER) are usually relatively high as opposed to the standard of care. Several of the new orphan disease drugs, for example, are not reimbursed by public payers such as the National Institute for Health and Clinical Excellence (NICE) in the UK and the Pharmaceutical Benefits Advisory Committee (PBAC) in Australia.[23,24] Furthermore, considering the price erosion that occurs alongside patent expiration in several therapeutic areas due to the increased availability of generic treatment options, it becomes even more difficult to obtain the reimbursement listing approval from HTA Agencies.[25] As a result, R&D investments may become unattractive in certain therapeutic areas.

In order to ensure and enhance innovation in such markets while focusing priorities on an efficient allocation of resources, new criteria should be considered in the decision-making process for the reimbursement of innovative treatments. Some authors have highlighted the relevance of thinking beyond short-term cost reductions to also consider disease-specific characteristics in the decision-making process.[14] Others have proposed abandoning the "out-of-date" and unjustifiable $50,000 ICER threshold and promoting the use of thresholds that vary across payers, populations, and even procedures.[26] Another avenue of research is the adaptation and enrichment of the ICER calculation in function of the context of the specific therapeutic area. This paper proposes a new methodological aspect that could be considered in the cost-effectiveness analysis of certain drugs: the use of the "patented price" of the comparator after its patent expiry in the ICER calculation in order to promote innovation, particularly in therapeutic areas with unmet medical needs. In the following sections, we demonstrate that even promising drugs will face challenges in showing attractive ICERs as a result of recent changes in generic prices. We will also discuss the relevance of using the proposed approach in order to promote innovation using a practical example involving chronic myeloid leukemia (CML) treatments.

## RATIONALE

### The Impact of using a Fixed Acceptability Threshold on Pharmaceutical Innovation

In the established health economics literature, cost-effectiveness analyses are interpreted using an incremental cost-effectiveness ratio (ICER) or an incremental cost-utility ratio (ICUR) between two comparators.[16] The ICER is obtained as follows:

$$ICER = (Cost_{\ New\ Drug} - Cost_{\ Comparator}) \ / \ (Benefit_{\ New\ Drug} - Benefit_{\ Comparator}) \quad (1)$$

where Cost is defined as the total costs of the drug including acquisition costs and all other costs (administration, monitoring, savings from avoiding the disease, etc.) and Benefit is defined as the drug benefit in terms of quality-adjusted life years (QALY)

There is a direct causal relationship between the HTA and both the market price of a certain drug and its ROI.[27] In a setting where HTA agencies apply a fixed ICER acceptability threshold as an important reimbursement listing criterion, the ICER of an innovative drug will need to be equal to or less than the ICER threshold to gain public listing:

$$ICER_{\ Threshold} \approx ICER_{\ New\ drug} = (Total\ Cost_{\ New\ drug} - Total\ Cost_{\ Comparator}) \ / \ (Benefit_{\ New\ drug} - Benefit_{\ Comparator}) \quad (2)$$

where Total Cost is defined as the total costs of the drug including acquisition costs and all other costs (administration, monitoring, savings from avoiding the disease, etc.) and Benefit is defined as the drug benefit in terms of quality-adjusted life years (QALY)

Therefore, if "all other costs" are assumed to be the same for the new agent and its comparator, the following equation applies:

$$\text{ICER}_{\text{ Threshold}} \approx \text{ICER}_{\text{ New drug}} = (\text{Aqu.Cost}_{\text{ New drug}} - \text{Aqu. Cost}_{\text{Comparator}}) \ / \ (\text{Benefit}_{\text{ New drug}} - \text{Benefit}_{\text{ Comparator}}) \quad (3)$$

where Aqu. Cost is defined as the drug acquisition cost and Benefit is defined as the drug benefit in terms of quality-adjusted life years (QALY)

Consequently, the cost of the comparator has an important impact on the acquisition cost of a new drug. Under a fixed ICER threshold, the incremental acquisition cost of a new drug is expected to be proportional to its incremental benefit over the comparator.

$$Incremental\ cost\ of\ a\ new\ drug\ (\%) = Incremental\ benefit\ of\ a\ new\ drug\ (\%) \quad (4)$$

Although the HTA process using cost-effectiveness thresholds facilitates the allocation of scarce resources, it creates a barrier to innovation after patent expiry of the standard of care in specific therapeutic class.

The drug price decrease associated with patent expiry theoretically diminishes the ICER acceptability threshold in the drug class and therefore, increases the ICER of the new drug.

$$ICER_{New\ drug} = (Aqu.\ Cost_{New\ drug} - Aqu.\ Cost_{Comparator}) * (1 - \%\ price\ decrease) / (Benefit_{New\ drug} - Benefit_{Comparator}) \quad (5)$$

where Aqu. Cost is defined as the drug acquisition cost, Benefit is defined as the drug benefit in terms of quality-adjusted life years (QALY) and % price decrease is defined as the percentage reduction in drug cost after patent expiry

Under a fixed acceptability threshold, the incremental benefits of the new drug need to be at least the same magnitude of the price drop of the comparator after its loss of exclusivity (LOE) in order to set the same initial price of the patented comparator. For example, if a cancer drug becomes generic and the generic versions are 75% less costly than the branded drug, the subsequent innovative drugs need to display relative incremental benefits 75% greater than the comparator to be considered cost-effective. To maximize profits and ensure market access, the optimal price of the new agent can be easily calculated as follows:

$$Cost_{New\ drug} = ICER_{Threshold} * Incremental\ benefit_{New\ Drug} + Cost_{Comparator} * (1 - \%\ price\ decrease) \quad (6)$$

where % price decrease is defined as the percentage reduction in drug cost after patent expiry and Incremental benefit $_{new\ drug}$ is defined as the incremental benefit of the new drug in terms of quality adjusted life years (QALY) as opposed to the comparator (i.e. Benefit $_{new\ drug}$ – Benefit $_{comparator}$)

As a result, some promising drugs need to be priced even lower than the patented price of their "less-effective" older comparators, which translates into a lower ROI. R&D investments in certain therapeutic classes become less attractive for innovative pharmaceutical companies who foresee a lower probability of reimbursement listing at the launch price.

## Considering the Patented Price of Comparator Drugs into the Cost-effectiveness Calculation

One way to overcome this issue without affecting the well-established HTA process for drug reimbursement would be to incorporate complementary analysis or methods in the cost-effectiveness examination in consideration of the area of disease. In therapeutic areas with unmet medical needs, the ICER calculation of a new drug could include the comparator's patented price rather than the generic price, which would render the assessment process independent from change in healthcare pricing policies. By identifying the relevant disease areas, decision makers and HTA authorities could therefore convey the importance of investing in these diseases to manufacturers. From a manufacturer's stand point, it would represent incentives to allocate greater R&D efforts in these areas, thereby improving the likelihood of successive incremental innovation that may be translated into the improvement of treatments' clinical effectiveness. Adopting the proposed approach will result in the addition of the following complementary ICER calculation in the dossier:

$$ICER_{New\ Drug} = (Cost_{New\ Drug} - Cost_{Patented\ Comparator}) / (Benefit_{New\ Drug} - Benefit_{Patented\ Comparator}) \quad (7)$$

Where Cost is defined as the total costs of the drug including acquisition costs and all other costs (administration, monitoring, etc.) and Benefit is defined as the drug benefit in terms of quality-adjusted life years (QALY)

As opposed to the regular ICER calculation formula (equation 5), equation 7 will result in a lower incremental cost (numerator of equation 7) because the acquisition cost of the patented comparator is superior to those of the generic versions. Therefore, the new drug will have a lower incremental cost-effectiveness ratio (ICER), thus enhancing its chance of reimbursement listing.

Considering the specific context of a given therapeutic area and the associated unmet needs, this complementary analysis could provide the decision makers with information that may influence their decisions on whether or not to adopt a new drug. By accepting the coverage of a drug with an ICER above the acceptability threshold when compared to the generic comparator but under the threshold when compared to the branded product in a specific therapeutic class, decision makers will prioritize and reward R&D efforts for certain diseases. Ultimately, these incentives to innovate may produce potential societal gains and better treatment options for future patients.

This method is not applicable to every drug class; it is relevant in disease areas for which the generic versions of the standard of care have come into market when its effectiveness is far away from an Ed max state (i.e. disease area with considerable unmet needs when generics come into market) and the new drugs entering the market show incremental benefits yet have difficulty in achieving a level of benefits comparable to the magnitude of the price drop of the standard of care. It is particularly applicable in areas where there has been no innovation for a long time and where therapeutic alternatives are really inexpensive due to the price erosion that occurs alongside generic entries and drug price policies. In other therapeutic areas not perceived as being areas of unmet need, this proposal may also be presented as a complementary analysis or as a sensitivity analysis to provide additional information to decision makers about the cost-effectiveness of a new drug over the patented version of the standard of care.

## Practical Example

Chronic myeloid leukemia (CML) is a slowly progressing myeloproliferative disorder for which too many white blood cells are formed in the bone marrow affecting mostly adult and elderly individuals. Without proper treatment, the average survival rate is three to five years.[28] Although, there are several effective treatments in this therapeutic area, CML is still an incurable disease unless an allogenic stem cell transplant (SCT) is successfully undertaken.[29] However, due to the risk of transplant-related mortality, pharmacological treatments are a more desirable first-line therapy.[30] Tyrosine kinase inhibitors (TKI) have shown impressive results in clinical studies. Among those, imatinib (Gleevec), the first-line CML therapy, has been associated with 7.03 QALYs, representing incremental benefits of 1.99 QALYs over interferon-α (IFN-α) and 2.04 over hydroxycarbamide.[31] The associated incremental cost-effectiveness ratios were respectively £26,180 (≈$44,300) and £86,934 (≈$147,005) per QALY.

Following previous drug reimbursement reviews at the national and provincial levels, imatinib (Gleevec) is presently reimbursed in every Canadian province as the first line treatment for CML. Its annual costs vary approximately between $36,000 and $56,000.[32] Considering an average survival period of 96 months, we estimated the lifetime acquisition cost of imatinib (Gleevec) to be $368,000 and other associated costs to be $108,230 in Canada.[31] In March 2013, imatinib (Gleevec) is expected to lose exclusivity[33], resulting in a 50 to 75% price decrease depending on the price policies in place in each province. When applying the rationale presented in this paper, if generic imatinib costs 25% of the original price (annual cost ≈ $11,500; lifetime cost ≈ $92,000), a new drug for the first-line treatment of CML would need to demonstrate significantly higher benefits to be listed at the same initial price of imatinib before loss of exclusivity (LOE) ($46,000 annually). In fact, if we assume all other costs to be the same for the new drug (lifetime costs ≈ $108,230), it would need to show an approximate 75% increase in clinical benefits (12.55 QALYs) where the ICER threshold is assumed to be $50,000 or a 50% increase in benefits (10.71 QALYs) where the ICER threshold is $75,000 in order to become cost-effective (equation 8). Considering that treatments are rarely curative and only extend life or improve the quality of life, such a requirement for improvement presents an insurmountable challenge to industry. Taking into account that imatinib had an incremental benefit of 1.99 QALY over the older non-specific treatment INF-α, it would be ambitious to expect a new drug to show incremental benefits of 3.5 to 5.5 QALY over imatinib or other effective new-generation treatments.

$$ICER_{New\ CML\ drug} = (\$476{,}230 - \$200{,}230) / (Benefit_{New\ CML\ drug} - 7.03\ QALY) \quad (8)$$

At $50,000 and $75,000 ICER acceptability thresholds, an innovative CML treatment would need clinical benefits of 75% (12.55 QALY) and 50% (10.71 QALY) greater than imatinib, respectively, after its loss of exclusivity.

Figure 1 highlights the impact of generic drug price regulations on the cost-effectiveness of innovative drugs. Assuming an innovative CML drug would possess an incremental benefit of 1.99 QALY over imatinib and would have the same price of the branded version of imatinib (Gleevec) (monthly costs of $3,833), the cost-effectiveness assessment would approximately result in a ICER of $44,873 as opposed to the innovative version of imatinib (Gleevec) before its LOE. After patent expiration the cost-effectiveness calculation of the same innovative therapy would result in ICERs of $137,000 per QALY in jurisdictions where generic drug prices are 50% of the patented drug price and as high as $196,000 per QALY where generic drug prices are 18% of the patent drug price (figure 1). This example highlights that even though the ICER of an innovative CML therapy would be below a $50,000 acceptability threshold when compared to the innovative version of imatinib, thus demonstrating cost-effectiveness, the patent expiry of the comparator is more likely to increase drastically the ICER of this promising innovative therapy and reduce its chance of drug reimbursement by public drug plans.

**Figure 1.** Impact of Generic Drug Price Regulations on the Cost-effectiveness of Innovative Therapies

The example of an innovative CML drug with incremental benefits of 1.99 QALY (extra survival of 18 months) over imatinib and the same price of the branded version of imatinib (Gleevec) (monthly costs: $3,833).

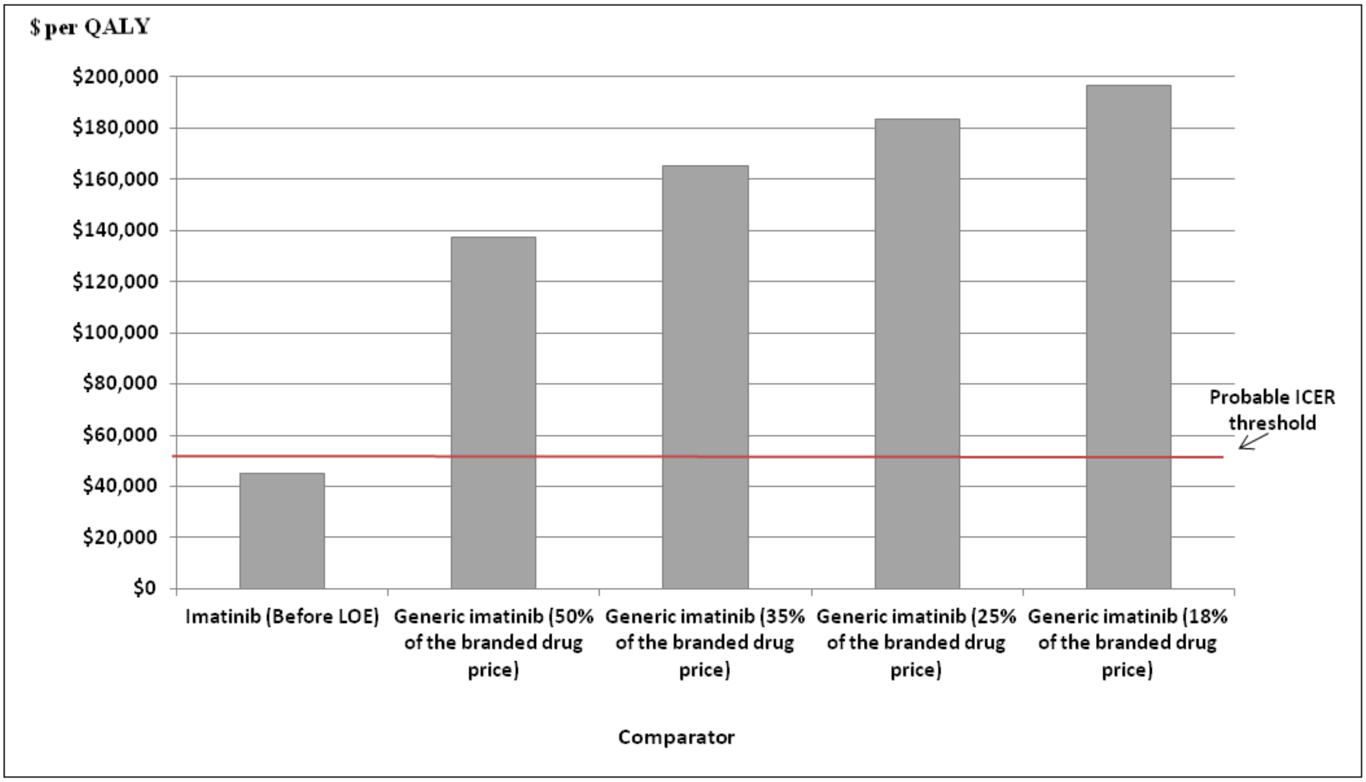

ICER: Incremental cost-effectiveness ratio; LOE: Loss of exclusivity; QALY: Quality-adjusted life years

Therefore, under the current model, Gleevec's patent expiry is expected to reduce the return on investment of new CML treatments and potentially affect the incentives to innovate in this therapeutic area. This highlights that the recent generic price regulations are not only affecting generic firms, but also may affect the level of R&D efforts innovative firms would allow for a specific therapeutic area. After the commercialization of generic versions of imatinib, considering its patented price in the ICER calculation would be a way to support innovation in the CML therapeutic area if decision makers perceive a high level of unmet needs.

## DISCUSSION

Several authors have raised the potential negative impacts of recent price regulations and the use of fixed thresholds for drug reimbursement purposes.[9,14,34] Using the example of hypertensive drugs, Refois Camejo et al.[25] have demonstrated that if the current cost-effectiveness appraisal scheme had been used initially for drug reimbursement decisions of the drugs commercialized between 1998 and 2008, a number of effective drugs might have never been adopted.

Considering the actual medical and pharmaceutical context and the associated innovative and highly specialized treatments, the traditional decision-making process and the statistical and economic methods need to be adapted to reflect contextual changes. Recent drug reimbursement decisions counteract important trends in publicly-funded research fostering innovation in personalized medicine. In such specialized therapeutic areas, ICER thresholds should be adjusted and in the case where the comparator prices were decreasing, the associated savings should be reinvested to fund innovation (i.e. increasing relative willingness to pay). We believe it is the ideal time to update current HTA practices to incorporate various criteria that may positively influence the traditional cost-effectiveness appraisal methods. Multi-criteria decision analysis (MCDA) and the associated specialized statistical methods are entirely applicable and needed to reflect actual contexts.[35]

The UK Department of Health, Pharmacy and Industry Group has recently shown interest in a modified decision-making process by initiating a consultation on a value based approach to the pricing of branded medicine (36). In their call for consultation, the authors highlight the importance of the pharmaceutical industry in the UK and propose more flexibility by weighting the ICER threshold in regards to the value of the innovation in the disease area. Analogies could be made with Canada, where cost-effectiveness appears to play an important role in decision-making.[5] While it is important to maintain incentives for pharmaceutical firms to invest in R&D, it remains unclear how this value based approach of pricing would be applied.

By integrating the method proposed in this paper into the cost-effectiveness assessment, one could bring forth additional information to decision makers without disrupting the well-established HTA process. In addition, it would allow more flexibility in the drug reimbursement decision making, especially in areas of unmet medical need. This proposal evidently represents only one element among many others to improve the decision making process. In addition, it represents one perspective from the pharmaceutical industry aiming at bringing forth further constructive debates, and therefore, it may have certain limitations. The theoretical rationale and the example presented in this paper do not take into consideration clinical nuances specific to certain disease areas. In addition, the notion of clinical effectiveness ceiling (Ed max) represents a theoretical ideal used in this article to describe the potential level of unmet medical need in a given disease area and may not easily be calculated by decision makers and HTA agencies. However, unmet medical need can be assessed through different means such as medical practitioner opinions and patient inputs. Future studies should further evaluate the health and financial impact of using this complementary method inspecific therapeutic fields.

## CONCLUSION

Throughout this paper, we have highlighted that the use of the patented price of generic drugs in the ICER calculation may be a good strategy to ensure innovation in areas of unmet need. As a complementary analysis to add to the dossier, it will still allow the use of different ICER thresholds as reference points for decision-making and decision makers would have the possibility to decide when it is relevant to use this information as an important criterion or only look at it as a complementary result. By doing so, decision makers and HTA authorities could therefore convey the importance of investing in these diseases to manufacturers. The relocation of R&D funding to areas of unmet medical need would most likely be detrimental to therapeutic classes where there are several treatment options providing sufficient clinical effectiveness.

## ACKNOWLEDGEMENTS

The authors would like to thank Rolina Van Gaalen and Pierre-Alexandre Landry for their helpful comments at different stages of this research. The usual disclaimer applies.

### Conflict of Interest Declaration

Pierre-Alexandre Dionne, Farzad Ali and Mendel Grobler are employees of Pfizer Inc., but ideas expressed in this paper are entirely those of the authors and do not necessarily represent the views of Pfizer Inc. No sources of funding were used in the preparation of this article.
